# Human-centered assessment of robotics and exoskeletons in construction industry

**DOI:** 10.3389/frobt.2025.1645150

**Published:** 2025-12-02

**Authors:** Susanne Niehaus, Rebecca Erlebach, Patricia Helen Rosen, Sascha Wischniewski

**Affiliations:** Federal Institute for Occupational Safety and Health, Unit Human Factors and Ergonomics, Dortmund, Germany

**Keywords:** human-robot interaction, construction, robotic systems, workplace automation, user expectation, human factors, usability

## Abstract

**Introduction:**

Robotics and wearable systems are increasingly being discussed as potential solutions to address the physical demands, skill shortages and safety risks faced by the construction industry. However, their successful implementation hinges not only on technical feasibility, but also on their alignment with real working conditions. This article examines how interactive robotic systems and exoskeletons are experienced by construction workers by integrating macro-level data from European and national surveys with micro-level insights from pilot studies.

**Methods:**

Five large-scale European surveys were analysed and combined with data from four pilot studies involving 37 workers interacting with three robotic prototypes and one upper-body exoskeleton. Quantitative data included usability, workload, interaction principles and affinity for technology. Qualitative feedback was obtained through open-ended responses.

**Results:**

A set of guidelines for a human-centred approach to inform policy were derived, offering practical guidance on designing and deploying interactive robotic systems that are functional, safe, acceptable and effective in changing work environments.

**Discussion:**

The observed challenges highlight the gap between the early stages of system design and the realities of dynamic construction work, emphasising the need for a participatory, human-centred development approach. The findings suggest that a human-centred approach is essential for emerging technologies to be functional, safe, acceptable and effective in changing work environments.

## 1 Introduction

The construction sector is one of Europe’s key economic sectors, accounting for 9% of the EU’s gross domestic product and employing more than 18 million people (European Agency for Safety and Health at Work, 2024). Despite its economic relevance, the sector faces persistent challenges, including physically demanding tasks, increasing skill shortages and complex safety standards (Irastorza et al., 2020). In response to these issues, technological innovations, particularly advanced robotics and wearable systems such as exoskeletons, have gained attention. These emerging technologies not only hold the promise of physical relief but have the possibility to fundamentally change the reality of work on construction sites with far-reaching consequences for work organization, psychosocial conditions and occupational safety (Nnaji and Karakhan, 2020; Maali et al., 2024).

However, despite their potential, digital technologies remain underutilized in the construction industry. For example, only 30% of surveyed enterprises in Belgium reported awareness of new digital technologies, and just 5% implemented them. This shows that despite its significance, construction is lagging behind other sectors in the race to digitalize (Fundación Laboral de la Construcción, 2020). The majority of advanced robotic systems, regardless of industry, currently aid in physical tasks. In particular, tasks such as heavy lifting, transporting objects, and assisting with strenuous movement are common (Rosen et al., 2022). These tasks typically fall under the so-called “3D” (dull, dangerous, dirty) jobs. This is often in line with manual material handling (MMH) tasks and exposure to forced postures. Both are frequently found on construction sites as shown in major surveys (Cavet, 2024; Irastorza et al., 2020). Maali et al. (2024) emphasize the growing importance of automated systems in mitigating human error and promoting safety compliance. Additionally, robotic systems and wearables that either distance humans from or support them during hazardous tasks could reduce the risk of long- or short-term injury by sharing biomechanical load and reducing fatigue (Kong et al., 2023).

Numerous studies have demonstrated that interactive robotic systems and exoskeletons can decrease physical strain, enhance task precision, and improve occupational safety (Gualtieri et al., 2021; Maali et al., 2024). In the context of industrial workplaces, particularly those engaged in manufacturing, these emerging technologies have been shown to reduce biomechanical load and enable ergonomic workplace design (Rosen et al., 2022; Sylla et al., 2014). However, the application of these technologies in unstructured, dynamic environments, such as construction sites, remains limited in both practice and research (Nnaji and Karakhan, 2020; Elprama et al., 2022). Although technical feasibility has improved tremendously, challenges such as technological complexity, environmental volatility, and limited user acceptance persist (Meyer et al., 2019). Particularly psychosocial risks such as low perceived control, time pressures, and unpredictable system behavior are associated with lower acceptance and even increased stress when workers have to interface with robotic systems (Lee and See, 2004; EU-OSHA–European Agency for Safety and Health at Work, 2022).

In addition to the previously mentioned barriers, the implementation of technology in the workplace must adhere to occupational safety and health principles, particularly the hierarchy of control. Organizational measures should be prioritized over technical solutions, such as robots, and personal measures, such as exoskeletons, should be the last resort (Schick, 2018). Some workstations require complex gestures, an accurate grasp, and dexterity. However, current interactive robotic systems still have limitations in terms of feasibility, perception, speed and flexibility. One way to address these limitations is to incorporate wearable solutions. Exoskeletons can provide helpful functions that fulfill industrial ergonomic needs, such as supporting postural loads and allowing flexibility in task selection (Sylla et al., 2014). Exoskeletons have been demonstrated to be perceived positively by workers, who expect them to alleviate physical strain and improve overall health, but they must be carefully integrated into workflows to ensure acceptance and effectiveness (Elprama et al., 2020; Elprama et al., 2022). Considering the immense physical demands of construction tasks, along with unstructured surroundings, the integration of robotics and exoskeletons has emerged as a potential solution for safer and more efficient workplaces (International Federation of Robotics, 2019).

To ensure the success of these technologies, it is crucial to understand and design for the humans interacting with the technology. The EN ISO 9214–110 standard on the ergonomics of human-system interaction (ISO 9214–110, 2020) is one source of guidance on the design of human-robot interaction. The standard contains seven relevant interaction principles: *Suitability for the task*, *Self-descriptiveness*, *Conformity with user expectations*, *Learnability*, *Controllability*, *Error robustness*, and *Suitability for individualization*. The revised version integrates the principle of *Individualization* into *Controllability* and introduces the new principle *User Engagement* (Heinold et al., 2025). Research has shown that potential future users of these systems perceive each interaction principle to be of varying importance: *C. with user expectations*, *Suitability for the task*, and *Controllability* are particularly important for interactions (Rosen et al., 2019). These key factors have been shown to improve trust and acceptance (Lee and See, 2004; Nomura et al., 2011). Level of expertise may also influence which interaction principles are prioritized by users (Niehaus et al., 2023). Nevertheless, all principles should be considered when designing the interaction. Another important theoretical framework is the Task-Technology Fit (TTF) model (Goodhue and Thompson, 1995), which posits that a technology will only have a positive impact on performance if its functionalities align well with the requirements of the tasks being performed. In construction, this means that interactive robots or exoskeletons must fit the *specific* physical and organizational characteristics of the job, including space constraints, workflow logic, and team dynamics. Poor usability, or a gap between system design and real work tasks, can cause rejection, misuse, or even increased risk (Goodhue and Thompson, 1995). This emphasizes the necessity of a human-centered approach in both the design and implementation of such technologies.

The involvement of workers in the design process from the outset has been demonstrated to enhance both usability and long-term acceptance (Niehaus et al., 2022). However, empirical studies investigating how workers experience robotic systems in real construction environments, especially during the early adoption phase, remain scarce. Large-scale surveys provide critical data on occupational risks, digitalization, and working conditions, but often lack the contextual detail necessary to understand worker-technology interactions in daily practice. Thus, there exists a clear incentive to bring pilot trials in real-world settings together with findings from extensive laboratory studies.

The objective of this paper is to address this knowledge gap by synthesizing insights from five large-scale European surveys with findings from real-world pilot studies. The paper seeks to address the following research question: How are interactive robotic and wearable systems experienced by construction workers, and what conclusions can be drawn from this for the human-centered design of future construction workplaces?

## 2 Materials and methods

This paper employs a dual methodological approach. Initially, data from large-scale European surveys are analyzed to provide insights into workplace conditions, technological transformations, and psychosocial stressors within the construction sector. This analysis offers a comprehensive understanding of the current state of the European construction industry and indicates future trends and areas for potential innovation. Secondly, the findings are supplemented with empirical results from real-world pilot studies conducted withing the framework of the HumanTech project (Human-centered Technologies for a Safer and Greener European Construction Industry, funding agreement no. 101058236). These pilot studies evaluate different interactive robotic systems and exoskeletons, focusing on overall user experience during human-technology interaction. Despite the exploratory nature of the current pilot studies, common approaches to structure our analysis were considered. Technological maturity, type of physical support, interaction modality and environmental robustness were identified as dimensions that enable cross-case reflection. This approach allowed for the identification of recurring challenges and key usability factors across the four prototypes, particularly between the robotic and exoskeleton systems.

### 2.1 Large-scale surveys on working conditions in the European construction sector

To gain a deeper understanding of current workplace conditions in the European construction industry, this paper draws on data from five large-scale European surveys conducted within the past 5 years. Each survey includes responses from individuals employed in the construction sector and covers aspects such as work environment, occupational safety and health, and the impact of digitalization. While the focus of the surveys varies, all provide valuable insights into working conditions and emerging challenges. An overview can be found in Table 1.

**TABLE 1:** Overview of large-scale survey data considered and analyzed.

Study/Survey	Total participants	Participants in construction	Focus/Topics	Source/Year
ESENER-3 (EU-OSHA)	45,420 (establishments)	3,267 (establishments)	Establishment survey on OSH management, psychosocial risks, digitalization, and worker involvement	European Agency for Safety and Health at Work (2023)
OSH Pulse survey (EU-OSHA)	27,242	2,313	Workers’ perception of OSH risks, psychosocial risks, WRMSDs, mental wellbeing, employer support systems	European Agency for Safety and Health at Work (2023)
GESIS working environmental report	24,402	2,339	German work environment, digitalization, physical workload, mental strain, overall job satisfaction	Own compilation
BIBB/BAuA employment survey 2024	20,006	998	Working conditions, qualification use, health, stressors, changing work conditions	Kaboth et al. (2025)

The largest dataset stems from the Third European Survey of Enterprises on New and Emerging Risks (ESENER-3, EU-OSHA–European Agency for Safety and Health at Work, 2022; Irastorza et al., 2020), which includes *N* = 45,420 participating establishments from 33 European countries, with *n* = 3,267 in the construction sector. Data collection was carried out using computer-assisted telephone interviews (CATI). The survey primarily focuses on OSH management, psychosocial risks, digitalization, and worker involvement.

The OSH Pulse survey (European Agency for Safety and Health at Work, 2023), commissioned by the European Agency for Safety and Health at Work (EU-OSHA), includes *N* = 27,242 participants from all EU member states as well as Iceland and Norway. Of those, *n* = 2,313 reported to be working in the construction sector. The OSH Pulse survey focuses on post COVID-19 pandemic working conditions as well as workers’ perceptions of OSH risks and psychosocial risks.

The GESIS Working Environmental Report (Cavet, 2024) was conducted in Germany, Ireland, Spain, France, Hungary, and Finland, comprising *N* = 24,402 respondents in total, of whom *n* = 2,339 were construction sector employees. This dataset provides a comprehensive overview of working conditions, including physical workload, mental strain, and environmental conditions.

The BIBB/BAuA employment survey (Kaboth et al., 2025; Lück et al., 2024) is a representative cross-sectional survey carried out by the German Federal Institute for Vocational Education and Training (BIBB) in collaboration with the Federal Institute for Occupational Safety and Health (BAuA). It includes *N* = 20,006 participants, with *n* = 998 working in the construction sector. Using CATI methodology, the survey captures information on working conditions and the associated stressors and related health complaints.

In total, the analysis draws on responses from *n* = 5,650 individual employees and 3,267 enterprises working in the construction sector across Europe. These data provide a comprehensive basis for examining working conditions, trends in digitalization and automation, as well as potential challenges and implications for OSH and employee wellbeing.

### 2.2 Pilot studies on user experience with robotic and wearable systems

**FIGURE 1:**
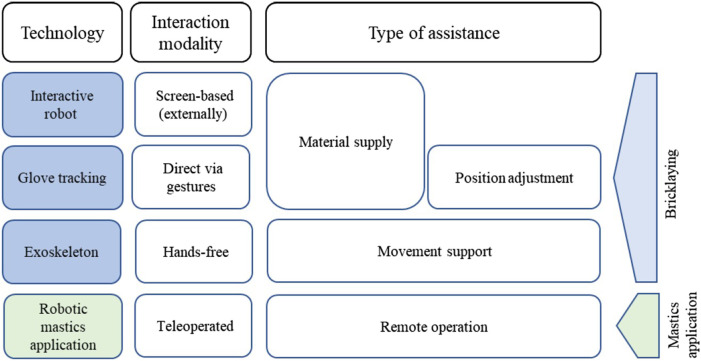
Overview of the four evaluated technologies.

The objective of the HumanTech project was to enhance safety, wellbeing, and productivity in the European construction industry through the development of interactive robotic systems and wearable technologies, including XR glasses and exoskeletons. Four pilot studies relevant for this work emerged within the scope of the interdisciplinary HumanTech project: three involved interactive robots and one an exoskeleton (Figure 1). An interdisciplinary approach was adopted in the design of the different prototypes, which were developed by multiple research institutions and industrial partners across Europe. To create conditions as close as possible to real-world construction environments, two construction tasks were selected for support by the technology under development. These tasks were bricklaying and applying mastics, both of which are frequent and physically demanding in the construction sector. Because the technologies were still in development, technological maturity varied considerable depending on the pilot study. While the exoskeleton was a market-ready product, the robotic applications remained at TRL levels below 5 (International Organization for Standardization, 2013). This variation in technological readiness shaped both the interaction requirements and control modalities applied in the studies. For instance, environmental robustness and sensitivity to site-specific conditions (e.g., uneven terrain or poor lighting) varied across systems. These variations were duly considered during the system deployment process and subsequently influenced the interpretation of user feedback.

Instead of a strict template, the instruments for the evaluations of the pilot studies were selected for each system based on the specific task constraints. Thus, the selection of evaluation methods was based on the unique features and limitations of each system, including its control logic, necessary user input, and robustness in relation to typical construction site conditions. Circumstantial reasons, such as questionnaire availability in different languages and the feasibility of the evaluation, as well as different planning timelines and the involvement of various project and research partners, explain the variations in questionnaires. Rather than proposing a single solution for all pilots, a toolbox was developed that contains various validated and reliable questionnaires. The purpose of this toolbox is to select the most appropriate questionnaire for each evaluation. This approach permitted a more flexible and feasible strategy to be adopted for each evaluation.

The aforementioned crucial constructs of interaction principles and Task-Technology Fit were considered in the process of technology development within the HumanTech project. The four pilot studies were conducted with workers interacting directly with the interactive robotic systems as well as an exoskeleton (Figure 1). To capture the multifaceted nature of HRI, the evaluation of their experience included the NASA-TLX (Hart and Staveland, 1988) to assess perceived workload across six dimensions (mental demand, physical demand, temporal demand, performance, effort, and frustration), each on a 21-point Likert scale. Another tool used was the System Usability Scale (SUS) (Brooke, 1996), a 10-item questionnaire using a five-point Likert scale (from strongly disagree to strongly agree) to give a global view of assessed usability. The USE (Lund, 2001) was also used. This questionnaire is a psychometric tool that assesses the user’s perceptions about a system’s usability and consists of four dimensions (*Usefulness*, *Ease of use*, *Ease of learning*, *Satisfaction*), which are measured on a seven-point Likert scale. It was used to evaluate usability and user satisfaction. In addition, the Affinity for Technology Interaction (ATI) scale (Franke et al., 2019) was used to provide an understanding of the individual’s predisposition towards novel technologies and includes nine items, rated on a six-point Likert scale. A shortened and adapted version of the IsoMetrics (Gediga et al., 1999), based on the Interaction Principles as described in ISO 9241–210:2019, was used to assess the overall quality of interaction. It consists of 12 items that are rated on a five-point Likert scale. Other measures were used for some technologies but are not taken into consideration for this article as they were either only taken for one evaluation or not relevant for the specific research focus addressed here. The complete measures can be found on Zenodo (HumanTech Consortium, 2025) and a short overview in Table 2.

**TABLE 2:** Overview of questionnaires used for each pilot study.

Measurement	Evaluated HumanTech technologies			
	Mastics application	Brick laying robot	Bricklaying robot – gesture control	Exoskeleton
NASA-TLX	X			
SUS	X	X	X	
USE		X	X	X
Interaction principles	X	X		X
Affinity for technology (ATI)	X	X	X	X
Technology specific questions	X		X	
Open-ended questions	X	X	X	X
Pain scale (NMQ)	X			

In addition to quantitative data, qualitative data was collected through open-ended inquiries. Here, the focus was on the participants’ overall assessment of the interaction and potential benefits and challenges they could foresee in the short- and long-term. Open-ended responses were collected after usability testing by filling out the questionnaires. A content-based method was followed without formal coding procedures. The text was subjected to a comparative analysis among the participants, with the objective of identifying emergent themes, recognizing recurring issues, and identifying suggestions that exhibit overlap. Feedback elements identified by two or more participants were taken to represent more user-oriented perspectives and were used to inform iterative system enhancement. Due to the exploratory nature of the study and limited sample size, no formal thematic or inferential statistical analysis was conducted.

Three of the four pilot studies were conducted at a project partner’s outdoor facility in Alcobendas, Spain. For these, all questionnaires were translated in Spanish and then re-translated for the analysis. Simple language and an efficient evaluation process were focussed on, anticipating a small sample size and potential communication barriers. One pilot study (Gesture-controlled bricklaying robot) was conducted in a laboratory setting in Trondheim, Norway. This pilot’s questionnaires were used in English. For all pilot studies, 1 hour was allocated per participant. The participants were first introduced to the technologies, then had the opportunity to use them and afterwards filled out the questionnaires.

To build the design guidelines from both, existing literature and findings from the field, a test economic, iterative approach was taken that integrated various sources of data rather than a formalized step-by-step process. Insights from relevant literature was complemented by qualitative answers to open-ended questions and quantitative results from described questionnaires. Therewith, they were integrated with mentioned literature to identify consistent themes and actionable findings. Due to the exploratory design of the study and the limited sample size, no statistical analysis or formal coding was performed. Rather, comments identified by more than one participant and validated through quantitative ratings and the literature were given precedence to guide system refinement and guideline creation.

#### 2.2.1 Mastics application

In construction, joints between planes, e.g., on the floor, are filled manually using mastics. This requires the worker to work in very unergonomic positions, which can have long-term health consequences as they are done repeatedly and often. To improve this workflow, a device was developed (Rasines et al., 2025) which can be teleoperated and includes a robotic arm that allows filling the joints remotely. To assess the teleoperated system with the robotic arm and haptic controller, a user evaluation with *N* = 10 workers was conducted. It included all mentioned measuring instruments, except the USE questionnaire, as an efficient evaluation process was needed for workers on site (HumanTech Consortium, 2025).

#### 2.2.2 Brick laying robot

Bricklaying is a routine and integral task in the construction industry and can be prone to accidents caused by manual handling. To mitigate this risk, the brick-laying robot was designed in order to assist bricklayers by carrying and handling bricks. The developed robot is able to pick up individual bricks from a pile and hand them over to the bricklayer. That means, that the bricklayer does not have to bend over and pick up each brick individually, leading to less load on the bricklayer’s shoulders and back. Instead, the bricks are handed to the bricklayers at a comfortable height. With that, the robot supports a more ergonomically correct body posture during the task. In this setup, the participant was in position at a brick wall. The robot was positioned perpendicular to the wall and the worker for safety reasons in case of robot malfunctions. After the start signal was given, the robot started to pick up individual bricks and handed them to the participant. The participant then took each brick and continued the brick wall using mortar and the handed bricks. To minimize waiting times between each brick handled by the robot, the participant also had their own pile of bricks to pick up and continue the wall. Each participant was handed at least three bricks by the robot. The evaluation included the SUS (Brooke, 1996), the USE questionnaire (Lund, 2001), the ATI Scale (Franke et al., 2019), as well as questions regarding the interaction principles and open-ended questions. Overall, *N* = 8 workers tried the technology and filled out the questionnaires.

#### 2.2.3 Gesture-controlled bricklaying robot

In addition to the brick-laying robot, another technology was evaluated for bricklaying. The focus here was on gesture control as an alternative interaction mode in HRI. In this pilot, participants were fitted with a glove, which enabled them to control the robot’s position for the handover using gesture control. The task was once again bricklaying with the robot picking up the bricks and handing them over to the participants. After laying eight bricks, the next row had to be started at the initial delivery location. For this, the robot’s location was controlled via glove tracking. Then the bricks are delivered with a reference to the newly defined position. This task was completed by *N* = 10 participants within a laboratory setup and subsequently evaluated. The evaluation included two parts: a pre-trial part (T1), which was completed prior to using the glove-tracking technology and a post-trial part (T2), which was completed after the participants finished the trials. In the pre-trial, participants were asked about their expectations of gesture-based robot control, which consisted of five items rated on a five-point Likert scale (1 = strongly disagree to 5 = strongly agree) while post-trial questionnaires included the SUS (Brooke, 1996), the USE (Lund, 2001) and open questions about the experience with gesture-based control to allow for a pre-post comparison.

#### 2.2.4 Exoskeleton

Bricklaying can be especially hard on workers’ bodies, in particular on the shoulders and arms when working at shoulder height or above. For these positions, exoskeletons offer support for workers. In this pilot, a passive, upper-body (shoulder) exoskeleton was worn by the participants while building a brick wall. Participants were asked to use some bricks placed next to the wall and mortar and continue building the wall at shoulder height. Each participant did the task with the exoskeleton on for about 15 min. Afterwards, the participants removed the exoskeletons and assessed their experience using the USE questionnaire (Lund, 2001), questions regarding the interaction principles as well as the ATI scale (Franke et al., 2019) and open-ended questions. A total of *N* = 9 participants used the exoskeleton and assessed their experience afterwards.

## 3 Results

### 3.1 Large-scale surveys: Data on working conditions and sectoral challenges in construction

Working conditions in the construction industry are marked by physical strain as well as environmental and demographic hazards, making it a particularly relevant context for investigating occupational health and safety as well as the role of emerging technologies.

The ESENER-3 survey, which collected information from *n* = 3,267 enterprises across Europe, shows that, in the construction sector, more than 70% of responding enterprises reported that their employees were required to lift or move heavy loads and perform repetitive hand or arm movements as part of their work. Other factors identified by enterprises as potentially contributing to worker exhaustion include constant noise, as well as exposure to heat, cold and wetness. The ESENER-3 findings corroborate that enterprises in the construction industry frequently face environmental and occupational risk factors affecting their workforce. Furthermore, the data indicate that 58.5% of workplaces are affected by noise, 67.5% by extreme temperatures and 60.8% by the risk of slips or falls. Notably, the risk of accidents involving machines, tools, or vehicles is reportedly higher in this sector than in others. According to a Eurostat article on workplace accidents in the EU, nearly a quarter (22.9%) of all reported accidents occurred in the construction sector (Eurostat, 2024). Since construction workers tend to be older than the average age of the population, with almost 60% aged between 50 and 65 (Müller et al., 2023), the combination of an ageing workforce, a high and demanding physical workload and challenging environmental conditions may contribute to this. Alongside their strenuous day-to-day work, construction workers are among the most exposed to physical risk factors (Irastorza et al., 2020). This situation poses serious challenges to health and productivity and to the retention of skilled workers, highlighting the need for robust and adaptable technologies. However, the level of digitalization remains relatively low. Although smartphones are widely used on site (82%), technologies such as robotic systems are among the least adopted forms of digitalization in construction. Only 3% of construction sector enterprises state that they use robotic systems, which is lower than in the manufacturing sector (9%). Wearable solutions are used almost as rarely: only 5% report using wearable devices such as smartwatches or data glasses.

Insights from the OSH Pulse survey by the European Agency for Safety and Health at Work (EU-OSHA–European Agency for Safety and Health at Work, 2022) reveal that construction workers (*n* = 2,313) experience considerable mental strain. A secondary analysis of OSH Pulse survey data provides further insights into digitalization and the use of digital technologies in the workplace. For the purpose of this analysis, the working conditions of construction and building sector employees using various technologies were examined. These technologies included wearable devices such as smart watches, smart glasses and activity trackers, as well as other embedded sensors; desktop computers; laptops; tablets; smartphones; and other portable computer devices; machines or robots that can think and make decisions; and robots that interact with users.

**FIGURE 2:**
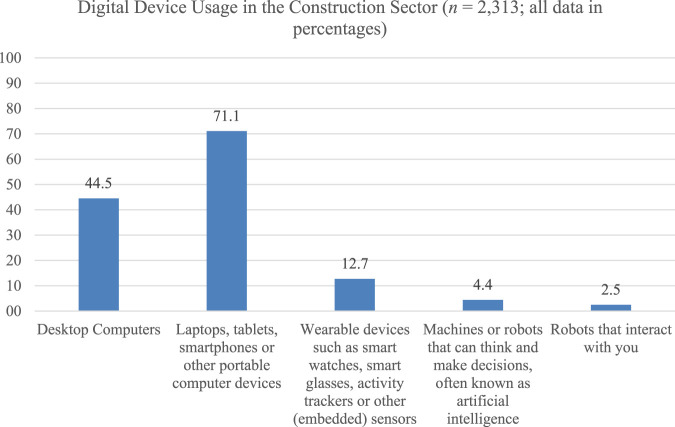
OSH Pulse Data on digital device usage in the construction sector. All data is presented in percentages.

Figure 2 shows that the most commonly used digital devices are laptops, tablets and smartphones (71.1%), followed by desktop computers (44.5%). When it comes to more innovative technologies, such as wearables and robotics, it is clear that there is potential for increased usage and development in the construction sector. Only 2.5% of employees reported using interactive robots. Wearables are somewhat more common, at 12.7%. However, it should be noted that this survey includes smart watches in the wearables category, and these have become much more common in general. The OSH Pulse data shows a similar trend to that seen in the ESENER survey. There, 3% of sampled construction employees reported using interactive robots, while 5% reported using wearable devices (EU-OSHA–European Agency for Safety and Health at Work, 2022).

**FIGURE 3:**
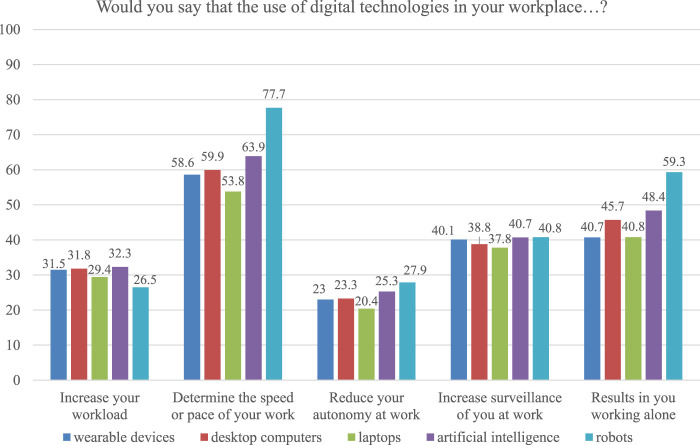
OSH Pulse Data: perceived impact of digital technologies in the workplace.

The OSH Pulse survey also includes questions about the perceived impact of technology usage in the workplace (Figure 3). Examining the data reveals definitive differences between the various devices. Robots are reported to increase the workload by a smaller amount than the other surveyed technologies (26.5%), while also determining the speed or pace of work more than any other technology (77.7%). This may be because robots usually work within a set cycle, meaning workers are more dependent on them to finish their work than on other technologies. This may also explain why robots are more likely to reduce workers’ autonomy (27.9%) and why the use of robots results in more frequent solo work (59.3%). In comparison, the impact of wearables appears to be less pronounced. They determine the speed or pace of work in fewer cases than robots (58.6%) and less often influence autonomy (23%). This may be because wearables do not directly intervene in work processes, instead influencing the workers themselves. This is also evident when asked if using wearables results in working alone, which is rated lower than with other technologies (40.7%).

The GESIS survey (*n* = 2,339) suggests that the less frequent use of innovative technologies such as wearables and interactive robots on construction sites may be due to the volatile and challenging ambient conditions, such as poor lighting and uneven terrain, that are often present. 70.2% report that they worked on building sites in the last week, which includes many different types of buildings and environments, such as hotels, factories, and private homes. These conditions pose challenges to the robustness of technologies and influence the way humans interact with technical systems (Cavet, 2024). Unlike more controlled environments, such as manufacturing, construction sites impose technical and environmental constraints that many emerging technologies are not yet equipped for. Consequently, many robotic solutions for the construction industry remain in the prototype stage (TRL <5), which not only limits their functionality, but also their perceived usability and acceptance by workers. This further complicates their implementation in real-world scenarios (Pracucci et al., 2023). Consequently, construction remains one of the sectors lagging behind in digitalization, despite the clear potential of emerging technologies to alleviate workload and improve safety.

Finally, the BIBB/BAuA survey provides additional insights into working conditions in the construction sector in Germany. It encompasses many different factors, including environmental, social, structural and organizational factors. Similar tendencies to those seen in other large-scale surveys were evident in the German BIBB/BAuA employment survey (*n* = 998): nearly half of all respondents in the construction sector stated that they regularly worked at the limit of their capacity. More than half of these respondents need to work quickly and lift heavy loads. Moreover, most workers find their jobs repetitive, and pain in their lower back, shoulders, and neck is common. Most workers perceive this as stressful: in total, more than a third stated that they are physically exhausted by their work (Lück et al., 2024).

### 3.2 Mastics application

Overall, system usability of the robot was considered as acceptable, with the majority of users rating the system as “good” or “excellent”. Only one person rated the overall system usability as “poor” (values under 51.7). The results for each interaction principles in relation to the robotic system are presented in Figure 7. The system’s *Error robustness* was rated highest (*M* = 4.9, *SD* = 0.3), indicating that the robot handles wrong, missing or incomplete user inputs well and allows to correct them. *Learnability* was also perceived as very good (*M* = 4.6, *SD* = 0.5), showing that users were able to easily discover system functions through exploration. However, the interaction principle *Self-descriptiveness* was rated the lowest (*M* = 2.9, *SD* = 1.3) which suggests that the robotic system does not provide clear and immediate feedback to the users at all times. This makes it hard for users to understand their current position in the workflow and the next steps required to achieve their goal. This lack of transparency is a crucial factor when it comes to diffusion of responsibility as fewer clear processes can lead users to incorrect task allocation during critical moments of the interaction, ultimately leading to hesitant responsiveness when the human should take the initiative which not only can pose a high risk on the worker and work process but also reducing accountability and engagement in the collaborative process. Additionally, the diffusion of responsibility may pose legal and organizational challenges.

The analysis of the qualitative data indicates that workers anticipate changes in their working task if working more regularly with this robotic system. The most commonly mentioned benefits include improved quality and efficiency as well as allowing for faster and more precise work. Additionally, many workers expect better physical ergonomics, reducing physical strain and promoting overall health by minimizing awkward postures and heavy lifting. It was further noted by some that automation would result in a safer work environment; however, the manner in which this would occur was not specified. Nevertheless, users have expressed concerns regarding the reliability, speed, and current maturity of the system. As pointed out in the answers, this may not reflect a fundamental rejection of the concept, but rather dissatisfaction with the trial setup. Although the perceived physical demands decreased, users mentioned some effort and frustration while working with the robotic system. This finding indicates that while physical exertion may be diminished, the system must also be designed in a human-centric manner to facilitate intuitive interaction throughout the work process, thereby enhancing overall working conditions.

The evaluation indicates that the system has strong potential to reduce physical strain and improve ergonomics by minimizing awkward postures and heavy lifting. Usability was rated mostly as good to excellent, with high scores for *Error robustness* and *Learnability*. However, the system’s low *Self-descriptiveness* hindered intuitive interaction and created uncertainty during task execution. While users acknowledged benefits such as improved quality, efficiency, and safety, they also expressed concerns about the system’s speed, reliability, and current level of maturity. Despite the reduced physical exertion, reports of frustration emerged, underscoring the necessity for a more intuitive and user-centered design to effectively support workers in practice.

### 3.3 Bricklaying robot

**FIGURE 4:**
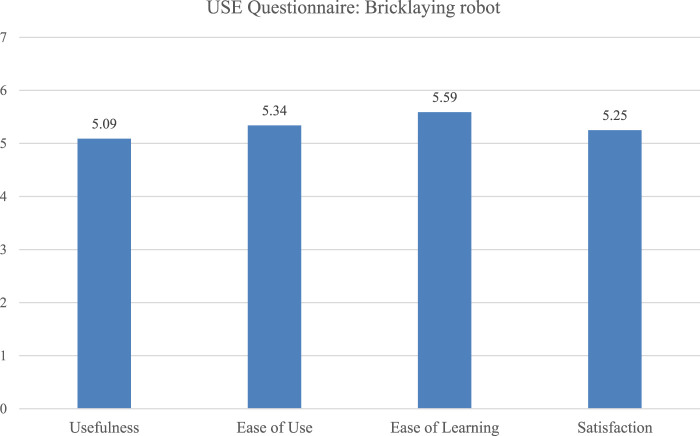
Comparison of four USE dimensions rated for the bricklaying robot.

The SUS scores ranged from 35 to 80, with an average of 59.7, which corresponds to “OK” rating of system usability. The wide variance suggests different levels of acceptance and perceived readiness of the system across users. Potential biases may have resulted from users witnessing technical errors during earlier sessions, which could have influenced their ratings negatively. The USE Questionnaire ratings were relatively balanced across the four dimensions, indicating general satisfaction, with slightly lower ratings for perceived *Usefulness* which might reflect technical problem during the study and are likely related to the robot’s currently limited speed and therewith real-world applicability (Figure 4).

The system was seen as suitable for the task (*M* = 4.38, *SD* = 0.7) and easy to learn (*M* = 4.25, *SD* = 0.83). *Controllability* (*M* = 2.38, *SD* = 1.11) and *Self-descriptiveness* (*M* = 2.88, *SD* = 1.17) were rated lowest. Since the robot was controlled externally, workers were not able to start or stop the application themselves. This lack of *Controllability* most likely impacted the user experience. *Self-descriptiveness* was probably undermined by limited visual feedback, especially under bright outdoor conditions.

The evaluation indicates that the system supports ergonomically favorable working conditions by handing over bricks at a comfortable height, reducing the need for bending and lowering strain on the back and shoulders. Usability scores varied widely, indicating mixed user experiences. As with the mastics application, the system was generally regarded as appropriate for the task and straightforward to master. However, it received low ratings for *Self-descriptiveness*. This negative rating taken together with the perceived lack of control over the interaction, the perceived usefulness was limited. These findings highlight that even if the core task is supported, shortcomings in system transparency and perceived control can create a gap between system design and user needs, potentially resulting in rejection (Goodhue and Thomson, 1995).

### 3.4 Gesture-controlled bricklaying robot

**FIGURE 5:**
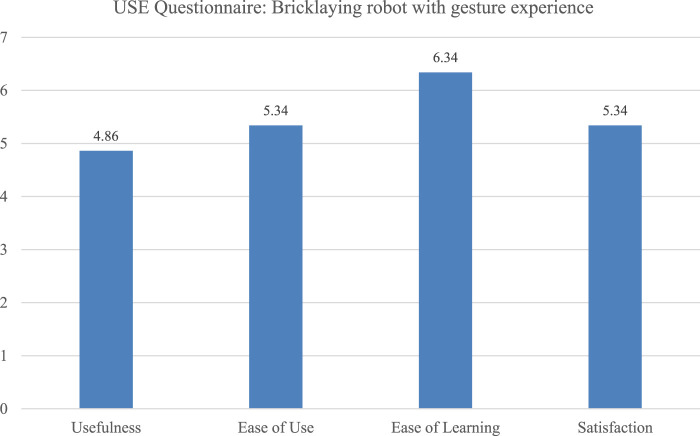
Ratings of the four USE Questionnaire dimensions for the bricklaying robot with gesture experience.

To compare participants’ expectations of controlling a robotic system with gesture with their actual experience a paired-samples t-test was conducted to compare mean scores pre (T1) and post (T2) trial. The analysis revealed a statistically significant decrease from T1 to T2, *t* (7) = 7.62, *p* < 0.001. Participants rated the gesture experience significantly lower (*M*_*Diff*_ = 1.08) after using the system, *d* = −2.69. Although expectations were high, the SUS scores after usage were very low for all participants, ranging from a score of 27.5–42.5. This is also visible in the USE scores where *Usefulness* was ranked lowest (*M* = 4.86, *SD* = 1.18). That indicates that gesture-based control was not perceived as an effective method for the given tasks. However, the system seemed to be easy to learn (*M* = 5.34, *SD* = 1.25), indicating that the gestures needed for the interaction were easy to perform (Figure 5).

Comparing the expectations with actual experience after working with the gesture control, it becomes visible that the expectations exceeded the actual usefulness. Participants rated the gesture experience significantly lower after using the system. This decline in perceived usability could be due to the physical effort required, recognition inaccuracies, or a lack of responsiveness in the system. However, the relatively high USE scores indicate that participants saw some applicability and understood the underlying interaction logic while the system was nor perceived as having met all needs for the task.

### 3.5 Exoskeleton

**FIGURE 6:**
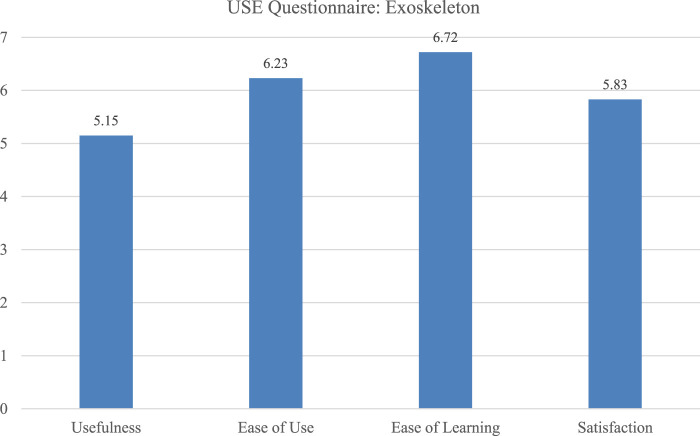
Ratings of the four USE Questionnaire dimensions for exoskeleton usage.

In this trial, workers rated all four dimensions of the USE questionnaire highly (*Ease of use* (*M* = 6.23, *SD* = 1.08), *Satisfaction* (*M* = 5.83, *SD* = 1.42), *Ease of learning* (*M* = 6.72, *SD* = 0.66)) showing that the wearable system was very easy to introduce into the work setting, not needing a lot of training or reassurance during usage (Figure 6).

**FIGURE 7:**
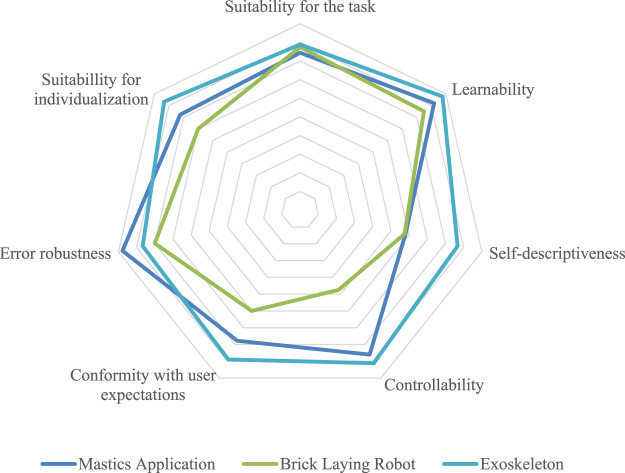
Comparison of interaction principles ratings including the original bricklaying robot, the mastics application and exoskeleton.

This positive feedback continued in the interaction principles rating as can be seen in Figure 7. All workers, except for one, stated that they would rather work with the exoskeleton in the future than without it. This is an indicator for a good task-technology fit. In the open questions, recurring elements that were reported by workers are the improved posture as well as less needed strength. In the long-term, the workers stated they see the potential of exoskeletons to reduce injuries, work-related musculoskeletal disorders (WRMSDs) and therewith promote health in general. At the same time, workers were sceptic about wearing the exoskeleton for a complete shift as they were not sure if it would be comfortable. 1n this regard, participants also raised concerns about the fit of the technology for unstructured environment like the construction site. However, the wearable system was seen as mostly effective and positive due to the reduction of stress on the body during the task.

The results indicate that the exoskeleton was easy to integrate into the workflow and required little to no training. Most participants preferred working with the exoskeleton, suggesting a strong task-technology fit. Workers recognized the long-term potential to reduce injuries and support musculoskeletal health. Despite mentioned reservations, the system was seen as effective in reducing physical stress during the task. The high maturity of the system most likely played a role in its positive evaluation.

## 4 Discussion

The construction sector remains a physically demanding work environment with frequent environmental changes and dynamic work situations. This leads to a high number of injuries and WRMSDs. Because of these challenges and rough working conditions, innovations in technology have come into focus to potentially improve wellbeing, safety, and productivity of work on site. In order to fulfill their full potential, such technologies must not merely function but also be usable, acceptable, and actually tailored to workers’ needs. That is, they must adhere to human-centric design. As research suggests, a high level of employee participation is needed to create technology that becomes embedded in actual workflows and improves everyday practice. The HumanTech project aimed to realize a first step in this direction by exploring the development and testing of a variety of emerging technologies from robotics that directly interact with the worker to exoskeletons. User participation was thus an integral feature of the project from start to finish. Despite the technology maturity of some of the tested systems, the feedback of real-life construction workers not only brought to the surface usability gaps but also made it possible to determine the main contextual limitations and unsatisfied needs early in the design process. In light of these observations and results of multiple large-scale surveys, design suggestions were formed that emphasize basic principles of Controllability, transparency, flexibility, and task-oriented design.

In the following sections, the pilot results are discussed in relation to national and European survey results, highlighting recurring patterns, complementing results, and system issues in the on-site deployment of robotic systems and exoskeletons. From this, design guidelines for emerging technologies are presented and discussed. The developed guidelines serve a two-fold function, serving as both practical, evidence-based guidelines for robotic systems and exoskeletons that account for the realities of the construction sector, and a model for subsequent evaluation and development cycles.

### 4.1 Comparing evaluation results with large-scale survey findings

The pilot studies provide valuable first-hand experiences regarding how construction workers perceive interactive robotic systems in the early stages of technological development. Including workers early on reduces the chance of a gap between system design and real work tasks. This gap can cause the rejection of the technology or increase the risk of injuries and misuse (Goodhue and Thomson, 1995). Furthermore, the acceptance and trust of workers towards the technology, and therewith, its effectiveness after implementation, depend heavily on understanding the system and its allocated tasks. Specific traits such as transparency and *Error robustness* are essential to this endeavor (Lee and See, 2004). This underlines the importance of a human-centered approach to designing and implementing such technologies.

Upon analyzing the above data, it was observed that most participants anticipated enhanced efficiency and task precision, reduced physical strain, and improved health and overall working conditions. All of the developed technologies were perceived as a potential aid for heavy physical labor, and most users evaluated the long-term operation of the systems under study quite favorably. Some users questioned the applicability of robotic systems to complex and non-repetitive construction operations. Others criticized the lack of transparency, reduced control, and uncertainty about how the system would behave. The results show that *Controllability* and *Self-descriptiveness* were persistent problems that most likely affected the usability of the system ultimately.

According to the OSH Pulse survey data, working with robotic systems is associated with greater time pressure, work overload, and general fatigue compared to other digital technologies. The same tendency can be seen in the German Digital Transformation and Changing World of Work survey (DiWaBe): those respondents who reported working with robotic systems also stated feeling significantly more time-pressured than other work groups (Arntz et al., 2020). The exoskeleton also showed these impacts, though to a smaller extent (EU-OSHA, 2023). Even though the systems tested did not generate time pressure, workers experienced less autonomy simply because they could not decide on the speed at which they worked, particularly when the robotic systems were externally controlled or when gesture control was not responsive. This was observed for both robotic systems. Problems, for example, frustration with gesture communication and absence of feedback from the bricklaying robot, are comparable to most common complaints in the OSH Pulse data: absence of user control and lack of knowledge about system behavior (European Agency for Safety and Health at Work, 2023). They also influence one another. Absence of confidence in the technology’s robustness induces a strong desire to be in control of the system. Both being seen as psychosocial risks linked to robotic technologies since it leaves space for diffusion of responsibility (Irastorza et al., 2019; European Agency for Safety and Health at Work, 2023). According to the DiWaBe data, working with robotic systems was linked to lower levels in perceived job control (Arntz et al., 2020; Hartwig et al., 2024). These results show the significance of paying attention to psychosocial factors within the design and implementation of interactive robotic systems. Emotional and mental demands may rise even when physical demands decrease. To avoid these obstacles, there has to be a constant human-centered evaluation of the implementation process and long-term effects. The DiWaBe survey data also corroborate the findings and underscore the importance of user-centered design. Workers who primarily engaged with robotic systems reported significantly lower physical strain, which confirms the potential benefits of these technologies (Arntz et al., 2020).

However, this does not necessarily translate into improved overall working conditions. Despite their advantages, adoption in the construction sector remains limited due to organizational resistance and the relatively low TRL of many applicable robotic systems (Pracucci et al., 2023).

Regarding the limited task-technology fit, contextual constraints on site might also have led to reduced *Satisfaction* regarding the trials with the bricklaying robot. These constraints may be indicative of structural barriers identified in representative workforce studies. Multiple barriers for the implementation of new technologies when working outside can be found in both, the GESIS and BIBB/BAuA employment data set (Lück et al., 2024; Cavet, 2024). Amplified noise can reduce the workers’ ability to react to sounds coming from the robot. Working under the sun does not only bring issues with high temperatures but reduces, as seen in the bricklaying robot pilot, the visibility of screens that might be of importance to a smooth interaction or even to understand potential problems and risks. Dirty surroundings, dust and uneven terrain can cause problems for the technology to function correctly and reduce the possibility to see, for example, light signals form the robot, as they might be covered. Qualitative data of the pilots shows that workers need long-term support when introducing emerging technologies on site since multiple concerns raised were focused on long-term usage and adaptability for changing task. In the qualitative parts of the BIBB/BAuA studies, employees state that an ongoing support and training when new technologies are introduced is needed, which also aligns with the observation regarding the need for long-term assistance. All in all, large-scale surveys underline the findings of the pilot studies and show that implementing emerging technologies on site faces several challenges. Nevertheless, they also indicate the key factors that need to be considered to ensure a safe and effective implementation process. Beyond the general patterns identified in large-scale surveys, the pilot studies revealed micro-level insights that only emerged during real-world testing. For instance, workers highlighted the difficulty of reading displays in bright sunlight and the unresponsiveness of gesture controls, both of which directly impacted their perceived autonomy and task efficiency. Such specific observations provide details that can be used to refine the interaction design of emerging technologies.

Taken together, the results demonstrate the need for user-centered design when developing and implementing emerging technologies such as exoskeletons and interactive robots. The issues reported emphasize the fact that even technically functional systems might not be aligned with the needs and working realities of users. It becomes particularly critical when the feelings of autonomy and responsibility are restricted by a lack of control or due to unclear system reactions. Systems must not only be easy to learn, but must also be able to be meaningfully and flexibly integrated into real work contexts. These insights form large-scale surveys taken together with first hand experiences from construction workers have been used to infer design guidelines for interactive robotic systems and exoskeletons which will be presented in the next paragraphs. Only if technologies are perceived as useful, controllable and understandable, they can contribute to a sustainable improvement in work processes and working conditions.

Based on these insights, the following operationalizable design principles for the integration of interactive robotic systems and exoskeletons on construction sites are proposed. By combining macro-level survey observations with micro-level evidence from real-world pilot deployments, actionable and context-sensitive guidelines were derived. These guidelines capture the general principles of human-centered design and translate them into concrete requirements tailored to the dynamic and demanding conditions of construction sites.

### 4.2 General design guidelines for emerging technologies

The development of the guidelines was initially based on relevant literature (Norman, 1986; Rosen and Wischniewski, 2017; Nomura et al., 2011; Elprama et al., 2022) and the existing standard EN ISO 9214–110 on the Ergonomics of Human-system interaction (ISO, 2020). This data was updated according to the findings from large-scale surveys and gathered data during the project (HumanTech Consortium, 2024; HumanTech Consortium, 2025) to contain both, the initial theoretical framework as well as user-centered design expectations and empirical evidence from the real world. In particular, user feedback gathered from the analysis of the emerging technologies impacted the guidelines refinement directly. Thus, the real specifics and constraints of the construction industry come into focus. This also enhances their sensitivity to evolving site circumstances. Although the development process did not follow a strict framework, it reflects a user-centered and context-sensitive approach appropriate for the explanatory nature of the project and the targeted construction environment. The recursive process ensured that the final design recommendations cater not only to theoretical considerations but also to actual working environment limitations and demands. From this, design guidelines for robotic systems as well as exoskeletons were derived that aim at improving *Controllability*, transparency and adaptability of emerging technologies on site. Therewith, human-centered design principles can be integrated into the development of technologies for an industry where usability, safety, and adaptability are essential to successful technology adoption.

### 4.3 Design guidelines for exoskeletons

The presented recommendations are based on Elprama’s framework for acceptance and usage (Elprama et al., 2022) but incorporate specific challenges in the construction sector that have been identified during the project. Overall, exoskeletons need to be suited for the specific task that is performed as well as for the target environment, that is, unstructured and changing environment outside. To ensure safety, exoskeletons should be compatible with protective clothing like helmets or gloves and their design should minimize the risk of tripping or falling. This is especially important in the construction sector as the risk for this kind of injuries is already higher than in other sectors. Feedback from the field emphasizes the importance of lightweight, unobtrusive exoskeletons. Workers reported that bulky designs restricted natural movement and slowed task execution. Similarly, the pilot studies emphasized the need for mechanisms that allow the exoskeleton to be easily put on and taken off, particularly for workers who alternate between multiple tools and tasks throughout the day. In this case, minimal adjustment time and high *Ease of use* create flexibility. This also includes the adaptability to different body types and individual needs. The robustness of the system is particularly important because of the harsh environmental conditions on site. Going beyond ergonomic interaction principles like *Suitability for the task*, *Suitability for Individualization* and *Learnability*, active exoskeletons need batteries that ideally should be long-lasting or easy to replace. Finally, the utilization of exoskeletons should always be voluntary, medically appropriate and based on thorough risk assessment, following the hierarchy of control and occupational safety and health standards (Elprama et al., 2020; Elprama et al., 2022).

### 4.4 Design guidelines for robotic systems

When introducing interactive robotic systems into the workplace, human factors including physical, cognitive and communicative aspects should be taken into account. As a result of the HumanTech project, multiple factors for the design of interactive robots for the construction sector have been identified. The construction sector’s characteristics include unique challenges, such as dynamic environments and demanding physical conditions. These challenges have implications for the technologies deployed, including the need for autonomy, user trust and adaptability. The pilot studies revealed several micro-level challenges that informed the design guidelines for robotic systems. For instance, workers frequently expressed frustration when the bricklaying robot failed to provide responsive feedback, or when externally controlled robots restricted their autonomy. These findings are further supported by survey results that highlight a perceived lack of job control in robotic environments (Arntz et al., 2020; Hartwig et al., 2024). The present study builds on these findings by identifying specific interface and control limitations that can erode trust and increase cognitive load. In order to mitigate such issues in general, the system must adhere to the human-in-control principle, ensuring decision-making authority of workers is left with the human (Niehaus et al., 2022). Moreover, robotic systems need to offer flexibility, e.g., changing working speeds, and be adjustable in line with the varying environmental conditions found on construction sites. This is especially important in the construction sector as weather conditions and the surroundings are changing on a day-to-day basis. Additionally, trust through reliable feedback, open data approaches, including data protection officers if present in the company and continuous training is equally important. In summary, a human-centered design approach should be prioritized to facilitate the integration of robotic systems into operational processes, while concurrently fostering safety, health, acceptance, and efficiency. Additionally, possible new emerging risks should be taken into account. For this, early employee involvement and iterative approaches to the introduction as well as long-term observations are crucial.

Taken together, the integration of macro-level survey evidence and micro-level pilot insights enables the development of operational, evidence-based principles, such as adaptive control mechanisms, robust and responsive feedback systems, and context-sensitive training. These principles offer concrete pathways to incorporate human-centered design into emerging technologies. By establishing usability, transparency and adaptability as tangible design priorities, these guidelines promote the adoption of safer, more efficient and more acceptable technology in construction environments. A comparison of the possible challenges that may arise when introducing interactive robotic systems on site and the proposed solutions can be found in Tables 3, 4.

**TABLE 3:** Design recommendations for interactive robots.

Challenges on site	Recommendation
Loss of control due to overly autonomous systems	Apply the “human-in-control” principle; humans retain decision-making authority
Fixed working speed	Enable variable speed settings to let workers adapt robot pace to task demands
Difficulty adapting to non-repetitive tasks	Conduct thorough risk assessment prior to implementation
Communication problems (e.g., speech in a noisy environment)	Adapt communication channels to the task and environment (e.g., gestures instead of speech in a noisy environment) and use multimodal communication channels
Lack of trust in the new system	Involve employees at an early stage and build trust through training and transparent system feedback
Negative attitude towards robots	Enable positive user experience through repeated interaction
Data protection concerns (e.g., cameras/sensor technology)	Create transparency: Provide information on what data is collected and what it is used for
Different user needs and experience levels	Design according to all seven interaction principles (ISO 9241–110), especially “*Suitability for the task*” and “*Controllability*”

**TABLE 4:** Design recommendations for exoskeletons.

Challenges on site	Recommendation
Physical discomfort or movement restrictions	Prioritize lightweight, ergonomic designs that allow full range of motion and minimize new physical strain
Difficult to put on and take off	Design exoskeletons that can be put on and taken off quickly, with minimal adjustment time, to support flexibility during task changes
Compatibility with protective equipment	Ensure compatibility with personal protective equipment (PPE) through design and testing in real work environments
Unsuitability for diverse body types	Incorporate adjustable components and individual customization options
Robustness in harsh environments	Build for environmental robustness with durable materials and protected mechanisms for outdoor conditions

### 4.5 Policy impact

Several pieces of EU legislation pertain to the use of technology in the workplace. The two most significant of these are the Machinery Directive 2006/42/EC (European Parliament and Council of the European Union, 2006), which will become the Machinery Regulation after January 2027, and the OSH Framework Directive 89/391/EEC (Council of the European Union, 1989). Both provide a legislative basis for the adoption of technologies and machines in general, as well as for AI-based systems and advanced robotics in particular. While the Machinery Directive mainly applies to products newly entering the EU market, the OSH Framework Directive is the fundamental legal framework for health and safety. It sets out several general principles for the prevention and protection of workers’ safety and health, such as avoiding risks, consulting workers and providing training. This directive aligns with the methodology for evaluating technology, which places a strong focus on early worker participation and evaluation. It also emphasizes that new technologies should be implemented in consultation with workers, as this benefits the selection of appropriate workplace equipment for worker safety and health. Furthermore, European strategies, such as the European Commission’s white paper on artificial intelligence (AI) and the European Social Partners Framework Agreement on Digitalisation set out key requirements and principles dedicated especially to AI. These fundamental principles include, for example, “Human agency and oversight”, meaning that a system should empower humans and allow them to make informed decisions. Other principles addressed in these strategies refer to transparency or fairness. Although the technologies addressed here are not AI-based, they can be considered as innovative and advanced work equipment. The application of these principles can therefore be extended to this type of technologies, especially as their connection with AI is steadily growing. Furthermore, it is clear, that claims for ethical and human-centered guidelines relating to such innovative technologies are not unfounded. The results collected in this work show that new and emerging technologies, such as those addressed in this work, do pose a risk to individual principles. Our research results contribute to policy-making actions by undermining shared values found in different activities on a European level. Thus, European regulation and strategies play an important role in determining the conditions under which such technologies are developed and deployed.

### 4.6 Limitations

While great efforts were made to maintain high scientific standards, a number of methodological remarks need to be taken into account when interpreting the results of the four pilot studies. First, the worker survey evaluations took place in the workers’ mother tongue and answers had to be translated for further analysis. The evaluations of the mastics application and the original bricklaying robot were conducted by workers who spoke Spanish, while the glove tracking alternative was tested by English-speaking users. In an effort to minimize potential distortions resulting from language differences, questionnaires and open-ended questions were translated into participants’ native languages prior to data collection and re-translated into English for analysis. These translations were performed by native speakers who are knowledgeable about the construction context and were also reviewed by researchers from the project team to achieve conceptual and contextual equivalence. Particular care was taken to preserve the meaning of key words in psychometric measures such as “usability”, “effort”, or “satisfaction”. Nevertheless, some subtle linguistic nuance was inevitably lost in translation. This was taken into account during qualitative analysis, which focused on recurring themes across multiple participants rather than isolated wording. In addition, the evaluations of the pilot studies included slightly different psychometric tools. This was done to have a more flexible and less constraint approach for each specific system. In most cases, the NASA-TLX was not taken into consideration as the complexity of the tasks were very limited. Given the brief interaction time and the relatively simple nature of the activity, participants were unlikely to experience meaningful levels of mental workload or temporal demand. The System Usability Scale (SUS), although a well-established measure, was not used in the evaluation of the exoskeleton, as it was a market-ready device. In the lab setting, focus was given to examining the basic technical functionality and user acceptability of gesture-based robot control under highly controlled conditions. Due to the exploratory nature and brief trial duration, a minimal set of measurement tools was utilized to reduce participant fatigue and focus on salient interaction mechanics.

Secondly, the technological maturity of the systems under evaluation varied greatly. At the time of the study, the robotic systems (mastics application, bricklaying robot, gesture-controlled robot) were still in the development or prototype stages. Therefore, their technological readiness level (TRL) was rather low. The used exoskeleton, on the other hand, was a commercially available, market-ready product with demonstrated reliability. This TRL imbalance likely influenced worker perception and rating of the systems: low-maturity prototypes are likely to possess functional limitations, bugs or a lack of robustness, all of which can negatively affect usability, trust, and acceptance. In contrast, the exoskeleton’s high TRL meant that a more stable and reliable user experience was delivered, and this could explain differences in user feedback (Figure 7).

Another factor that needs to be taken into account when interpreting the results is the limited interaction of workers with the systems. While these initial impressions are informative, they fall short in capturing long-term comfort, usability, and integration into daily work routines. Particularly with devices such as exoskeletons, issues when working a complete shift are not yet tested and need long-term field studies. Moreover, the evaluated technologies vary in their interaction modalities. The mastics robot was teleoperated using a haptic interface, the bricklaying robot was externally controlled or by a new embodied style of interaction (glove tracking) that allowed the worker to use gestures to control the position of the robotic arm. In contrast, the exoskeleton, functions passively, providing passive support to the upper body without requiring any interface interaction. The results indicate a wide spectrum of the applicability of robotic systems in construction and recurrent issues in the interaction. However, the different approaches to interact with the technologies most likely influenced the ratings of usability. Lastly, pilot sample sizes were small (between eight to ten workers) due to the limited availability of workers on-site, limiting generalizability. Nevertheless, the integration of quantitative ratings (SUS, USE, principles of interaction) and qualitative remarks through a combination of mixed methods enables the collection of multiple perspectives on users’ experience and expectations. The outcomes must be interpreted as exploratory data, providing initial knowledge for larger future studies with real-world environments and greater, more representative samples of users.

## 5 Conclusion

The paper explores how interactive robotic systems and exoskeletons are experienced by construction workers, and how the knowledge gained can be used to better design future construction sites for humans. Several core findings emerged across four real-world pilot trials. The evaluations performed within HumanTech confirmed the overall ability of these systems to reduce physical strain and facilitate ergonomic working practices. Although the robotic systems being tested had usability issues due to their low TRL, participants recognized their potential and, in most cases, looked forward to their long-term benefits. Workers acknowledged the potential of both robotic systems and exoskeletons to reduce physical strain and support safer ergonomic practices. However, they also identified several usability issues, including poor *Controllability* (e.g., fixed operational speeds or a lack of manual override), unreliable or limited feedback mechanisms, and design incompatibilities with dynamic outdoor conditions. Despite its market-readiness, the exoskeleton used raised questions about long-term comfort and fit within dynamic construction environments. These micro-level insights highlight the fact that technical performance alone does not guarantee acceptance or integration into daily workflows. This points to a challenging aspect of development since even if a system is theoretically very well-suited for a task, early prototypes will contain technical limitations, usability flaws or incomplete user interface designs. All of these issues can have a negative impact on user perception and acceptance. The findings also underscore the notion that technical maturity alone does not guarantee a high usability and task-technology fit, especially in complex and dynamic environments like construction sites. Instead, interaction quality, in particular, the degree of *Controllability*, *Self-descriptiveness* and adaptability, emerged as key drivers for the successful introduction of emerging technologies. This points to the significance of human-centered interaction design from the beginning of the development process. By incorporating feedback from actual workers from the start, not only did the project uncover design flaws but lay the foundation for an empirically tested, actionable set of design principles applicable to the construction setting.

Based on these results, this work advances the design and development of future robotic and exoskeleton systems by turning pilot evidence into practical principles. These principles prioritize intuitive interaction, task-environment alignment, and system adaptability, which are essential elements for ensuring safe, efficient, and acceptable technology usage in construction. As such, they provide a practical foundation for future innovation and responsible deployment of emerging technologies in this field. Furthermore, the paper makes distinct academic and practical contributions. From an academic perspective, it bridges the gap between macro-level evidence from large-scale European surveys and micro-level empirical findings, thereby refining the theoretical understanding of human-centered design in early-stage, low TRL-systems. It also emphasizes the requirement for TRL-sensitive evaluation frameworks that acknowledge the iterative nature of early-stage innovation and consider different interaction methods, such as teleoperation or gesture control. Known quantitative measurement tools tend to assume a minimum level of functionality and stability, which initial prototypes are not likely to possess. A more differentiated approach that accommodates early-stage innovation without penalizing systems for technical immaturity is therefore needed. The evaluation showed that traditional usability evaluation procedures may need to be reconsidered for low-TRL systems to allow a finer, context dependent judgment framework and therewith iterative improvement. Additionally, evaluations should account for whether the interaction is hands-free, screen-based, teleoperated, or direct. Thus, existing evaluation tools must be critically tested and adapted in their application to systems at early stages development. This ensures that feedback is contextual, meaningful, and supportive of iterative improvement rather than closing off early on to new technologies. Current usability assessment tools may not fully capture the nuances of such varied interaction modalities. Then, the importance of human-centered design becomes even more visible. As systems progress to higher TRLs, the design process for innovative technologies such as interactive robots or exoskeletons could benefit further from the support of Large Language Models (LLMs). As demonstrated by Lee and colleagues (2024), a general LLMs can be adapted to assist with the innovation and design of emerging technologies. Their initial case studies suggest that these automated, LLM-based approaches can effectively complement human-generated design solutions, thereby enhancing the innovation process as a whole (Lee et al., 2024).

Future research may integrate broader acceptance models such as the Technology Acceptance Model (TAM; Davis, 1989) or the Unified Theory of Acceptance and Use of Technology (UTAUT; Venkatesh et al., 2003) for deeper explanatory analysis. They were not applied here, as the systems were at early development stages and not yet suitable for full-scale deployment scenarios. In addition, cognitive ergonomics models such as Norman’s Action Theory (Norman, 1986) or Rasmussen’s SRK model (Rasmussen, 1983) may provide valuable theoretical frameworks for investigating user frustration with interaction found in trials. For instance, participants reported uncertainty while controlling the robot with gestures or lack of feedback in using telepresence systems. These issues may be explained as “gulfs of execution or evaluation” based on Norman. Similarly, Rasmussen’s SRK model could help account for cognitive overload when employees are forced to knowledge-based behaviour due to insufficient system transparency or feedback. Including such models in follow-up research could help distinguish more clearly between system design issues and user adaptation-related issues and thereby provide more effective design recommendations. Moreover, future studies should give importance to longitudinal field research that accompanies the deployment of emerging technologies for prolonged durations, allowing researchers to measure long-term usability, organizational integration under real-site conditions, and worker acceptance. A more formal comparative approach to emerging technologies in construction to analyse technological solutions, as found in Oladunni et al. (2025), can potentially provide dominant drivers of user adoption and integration success. This is particularly relevant in complex and high-risk domains such as construction. Such future directions can build directly on the foundations laid by the present work.

Overall, a comprehensive review of the current state of research and practice reveals significant gaps. This work addresses these current gaps by establishing the foundation for the integration of emerging technologies that are both functionally feasible and meaningful, acceptable and ultimately beneficial. This is a necessary step towards creating more efficient, sustainable and human-centered workspaces in the construction industry.

## Data Availability

The raw data supporting the conclusions of this article will be made available by the authors, without undue reservation.
